# Role of upregulation of the K_ATP_ channel subunit SUR1 in dopaminergic neuron degeneration in Parkinson’s disease

**DOI:** 10.1111/acel.13618

**Published:** 2022-04-20

**Authors:** Min Liu, Cui Liu, Xue Xiao, Shuai‐Shuai Han, Ming‐Xia Bi, Qian Jiao, Xi Chen, Chun‐Ling Yan, Xi‐Xun Du, Hong Jiang

**Affiliations:** ^1^ 12593 Department of Physiology Shandong Provincial Key Laboratory of Pathogenesis and Prevention of Neurological Disorders and State Key Disciplines: Physiology School of Basic Medicine Qingdao University Qingdao China

**Keywords:** ATP‐sensitive potassium channels, FOXA1, FOXA2, Parkinson's disease, sulfonylurea receptor 1 subunit

## Abstract

Accumulating evidence suggests that ATP‐sensitive potassium (K_ATP_) channels play an important role in the selective degeneration of dopaminergic neurons in the substantia nigra (SN). Furthermore, the expression of the K_ATP_ channel subunit sulfonylurea receptor 1 (SUR1) is upregulated in the remaining nigral dopaminergic neurons in Parkinson's disease (PD). However, the mechanism underlying this selective upregulation of the SUR1 subunit and its subsequent roles in PD progression are largely unknown. In 3‐, 6‐, and 9‐month‐old A53T α‐synuclein transgenic (α‐SynA53T^+/+^) mice, only the SUR1 subunit and not SUR2B or Kir6.2 was upregulated, accompanied by neuronal damage. Moreover, the occurrence of burst firing in dopaminergic neurons was increased with the upregulation of the SUR1 subunit, whereas no changes in the firing rate were observed except in 9‐month‐old α‐SynA53T^+/+^ mice. After interference with SUR1 expression by injection of lentivirus into the SN, the progression of dopaminergic neuron degeneration was delayed. Further studies showed that elevated expression of the transcription factors FOXA1 and FOXA2 could cause the upregulation of the SUR1 subunit in α‐SynA53T^+/+^ mice. Our findings revealed the regulatory mechanism of the SUR1 subunit and the role of K_ATP_ channels in the progression of dopaminergic neuron degeneration, providing a new target for PD drug therapy.

## Graphical abstract

The elevated expression of SUR1 subunit of KATP channels in nigral dopaminergic neurons was regulated by the transcription factors FOXA1 and FOXA2 at the early stage of PD.The upregulated SUR1 promotes its transmembrane transport and then causes the membrane hyperpolarization, which may promote the occurrence of cluster discharges mediated by NMDA receptors and affect the activities of dopaminergic neurons.

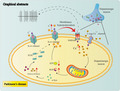

**Abbreviations**
AP: anteroposterior
ACSF: artificial cerebrospinal fluid
K_ATP_ channels: ATP‐sensitive potassium channels
α‐SynA53T^+/+^ mice: A53T α‐synuclein transgenic mice
^11^C‐CFT PET: 11 C‐2β‐carbomethoxy‐3β‐(4‐fluorophenyl) tropane positron emission tomography
DAT: dopamine transporter
DV: dorsoventral
Kir6: inward‐rectifier potassium channel 6
LBs: Lewy bodies
NMDA‐R: N‐methyl‐D‐aspartate receptor
ML: mediolateral
PFA: paraformaldehyde
PD: Parkinson’s disease
α‐Syn: α‐synuclein
SN: substantia nigra
SUR: subunit sulfonylurea receptor
TH: tyrosine hydroxylase
WT: wild‐type
FOXA1: forkhead box protein A1
FOXA2: forkhead box protein A2

## 1 INTRODUCTION

Parkinson's disease (PD), the second most common age‐related human neurodegenerative disease, is characterized by classical motor symptoms such as muscular rigidity, resting tremor, bradykinesia, and postural reflex disorders (Armstrong & Okun, 2020). The main pathological feature of PD is the selective loss of dopaminergic neurons in the substantia nigra (SN) accompanied by abnormal aggregation of α‐synuclein (α‐Syn), that is, Lewy bodies (LBs) (Vazquez‐Velez & Zoghbi, 2021). In addition to the genetic factors, environmental factors, aging, inflammation, oxidative stress, mitochondrial dysfunction, and abnormal iron metabolism, the selective activation of ATP‐sensitive potassium (K_ATP_) channels in dopaminergic neurons is also mediated the pathogenesis and development of PD (Abdelkader et al., 2020; Jiang et al., 2017; Nguyen et al., 2019; Trist et al., 2019; Zhang et al., 2018).

K_ATP_ channels are highly expressed in the SN, cerebral cortex, hippocampus, basal ganglia, and thalamic nucleus. These channels are located in cell bodies, axons, and presynaptic membranes (Moriguchi et al., 2018). The functional K_ATP_ channels, which are hetero‐octameric membrane protein complexes, are formed by four inward‐rectifier potassium channel 6 (Kir6; either Kir6.1 or Kir6.2) subunits and four sulfonylurea receptor (SUR; as SUR1, SUR2A, or SUR2B) subunits. They are ATP‐binding cassette subfamily members with regulatory activity (Li et al., 2017; Thomzig et al., 2005). Three types of K_ATP_ channels are expressed in the dopaminergic neurons: SUR1/Kir6.2, SUR2B/Kir6.2, and SUR1/SUR2B/Kir6.2 (Liss et al., 1999). The co‐expression of SUR1 and Kir6.2 in functional K_ATP_ channels is highly sensitive to metabolic inhibition in the SN (Yamada et al., 1997). Interestingly, the mRNA levels of the SUR1 subunit were found to be approximately twofold higher in dopaminergic neurons from patients with PD than in those from individuals in the control group, as determined by quantitative mRNA expression profiling techniques (Schiemann et al., 2012). In addition, previous investigations suggested that the activity of K_ATP_ channels promotes the switch from tonic firing to N‐methyl‐D‐aspartate receptor (NMDA‐R)‐mediated burst activity in vivo (Schiemann et al., 2012; Zweifel et al., 2009). Enhancement of K_ATP_ channel gating was necessary for burst firing in dopaminergic neurons in the SN. In addition, the dopaminergic neurons in the SN in awake PD patients exhibited high levels of burst firing activity, which accelerated the degeneration of dopaminergic neurons (Dragicevic et al., 2015; C. W. Lin et al., 2006; Liss et al., 1999; Liss et al., 2005). Given the abnormal expression and the role of K_ATP_ channels in patients with PD, it is highly important to clarify whether the expression of K_ATP_ channels in nigral dopaminergic neurons is changed in the early stage of PD and whether the K_ATP_ channel dysfunction is a cause or a consequence of the dopaminergic neuron degeneration.

The SUR1 subunit can be regulated by heat shock proteins, insulin, ghrelin, and protein kinase A in the peripheral nervous system (Chen et al., 2013; Heddad Masson et al., 2014; Yan et al., 2010). The SUR1 subunit was found to be upregulated at 1 hr and 8 hr after spinal cord injury, and this upregulation was alleviated by activation of growth hormone secretagogue receptor‐1a (Lee et al., 2014). In addition, FOXA1 and FOXA2, members of the forkhead/winged helix transcription factor family, were demonstrated to be able to bind to the SUR1 promoter directly in pancreatic beta cells (Cirillo et al., 2002; Jackson et al., 2010). However, little is known about the regulatory mechanism of SUR1 expression in nigral dopaminergic neurons.

In the present study, using mutant human A53T α‐Syn transgenic (α‐SynA53T^+/+^) mice, we aimed to investigate the changes in the expression and the role of K_ATP_ channels in nigral dopaminergic neuron degeneration in PD. To further explore the underlying mechanisms of SUR1 subunit dysregulation, we assessed the expression of FOXA1 and FOXA2 and their interactions with the SUR1 subunit. Our findings provide new insight into the involvement of K_ATP_ channels in the progression of PD.

## 2 RESULTS

### 2.1 The SUR1 subunit was upregulated in the SN in α‐SynA53T^+/+^ mice

**FIGURE 1:**
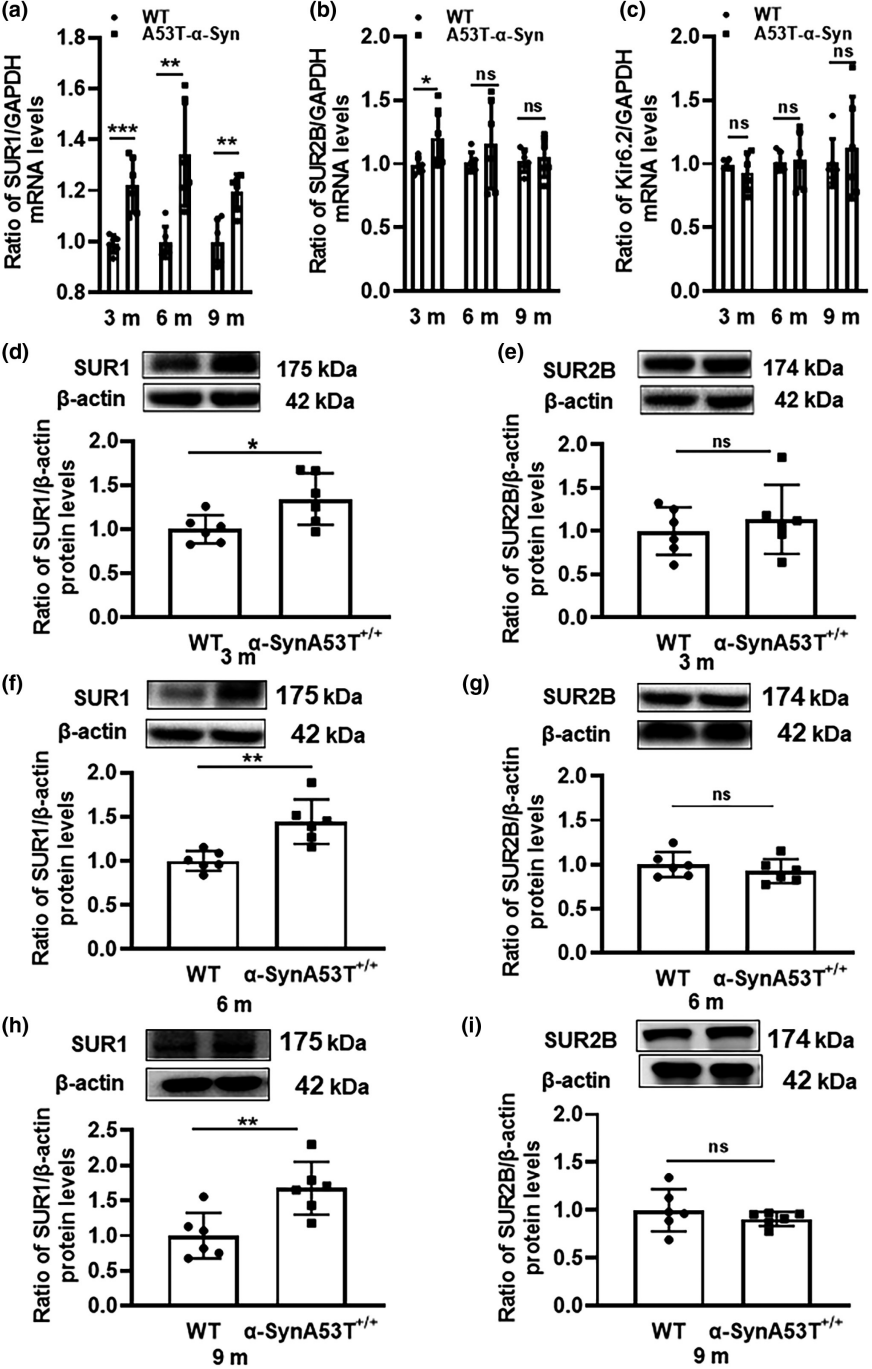
Changes in mRNA and protein levels of SUR1, SUR2B and Kir6.2 in the SN of α‐SynA53T^+/+^ mice at different ages. (a–c) The mRNA levels of SUR1, SUR2B, and Kir6.2 in 3‐, 6‐, and 9‐month‐old α‐SynA53T^+/+^ mice. The house‐keeping gene, GAPDH, was served as a standardized control. (d, f and h) The protein levels of SUR1 in 3‐, 6‐ and 9‐month α‐SynA53T^+/+^ mice (^*^ *p *< 0.05, ^**^ *p *< 0.01). (e, g and i) The protein levels of SUR2B in 3, 6, and 9‐month α‐SynA53T^+/+^ mice (^*^ *p* < 0.05, ^**^ *p* < 0.01, ^***^ *p* < 0.001)

To explore the changes in the expression of K_ATP_ channel subunits in α‐SynA53T^+/+^ mice at different stages, 3‐, 6‐, and 9‐month‐old α‐SynA53T^+/+^ mice were used. The mRNA levels of the SUR1, SUR2B, and Kir6.2 subunits of K_ATP_ channels in the SN were determined by RT–PCR. As shown in Figure 1a–c, the mRNA levels of the SUR1 subunit in the SN in 3‐, 6‐ and 9‐month‐old α‐SynA53T^+/+^ mice were increased by 23.41% (unpaired t test, *p *= 0.0005), 34.43% (unpaired t test, *p *= 0.0025), and 19.96% (unpaired *t* test, *p *= 0.0018), respectively, compared with that in age‐matched wild‐type (WT) mice. The mRNA levels of the SUR2B subunit were increased by 26.06% (unpaired *t* test, *p *= 0.0493) in the SN in 3‐month‐old α‐SynA53T^+/+^ mice. However, the mRNA levels of the SUR2B subunit in the SN in 6‐ (unpaired *t* test, *p *= 0.3375) or 9‐month‐old (unpaired *t* test, *p *= 0.7156) α‐SynA53T^+/+^ mice were not significantly different from that in age‐matched WT mice. Moreover, no significant difference in Kir6.2 expression compared with that in age‐matched WT mice was found in either 3‐ (unpaired t test, *p *= 0.3768), 6‐ (unpaired *t* test, *p *= 0.6203), or 9‐month‐old (unpaired *t* test, *p *= 0.5341) α‐SynA53T^+/+^ mice.

Subsequently, we determined the protein levels of SUR1 and SUR2B in the SN in 3‐, 6‐, and 9‐month‐old α‐SynA53T^+/+^ mice by western blot analysis. As shown in Figure 1d, f, and h, the protein levels of SUR1 in the SN in 3‐, 6‐, and 9‐month‐old α‐SynA53T^+/+^ mice were increased by 34.33% (unpaired *t* test, *p *= 0.0430), 44.53% (unpaired *t* test, *p *= 0.0150), and 67.53% (unpaired *t* test, *p *= 0.0076), respectively, compared with those in age‐matched WT mice. The levels of SUR2B in the SN in 3‐month‐old α‐SynA53T^+/+^ mice were not significantly different from that in age‐matched WT mice (unpaired *t* test, *p *= 0.5112), and the same result was found in 6‐month‐old SynA53T^+/+^ mice (unpaired *t* test, *p *= 0.3704) and 9‐month‐old SynA53T^+/+^ mice (unpaired *t* test, *p *= 0.3607) (Figure 1e, g, and i). Most importantly, both the mRNA and protein levels of the K_ATP_ channel SUR1 subunit were increased, which might influence the function of dopaminergic neurons in PD.

### 2.2 Changes in the firing activity of nigral dopaminergic neurons in α‐SynA53T^+/+^ mice

**FIGURE 2:**
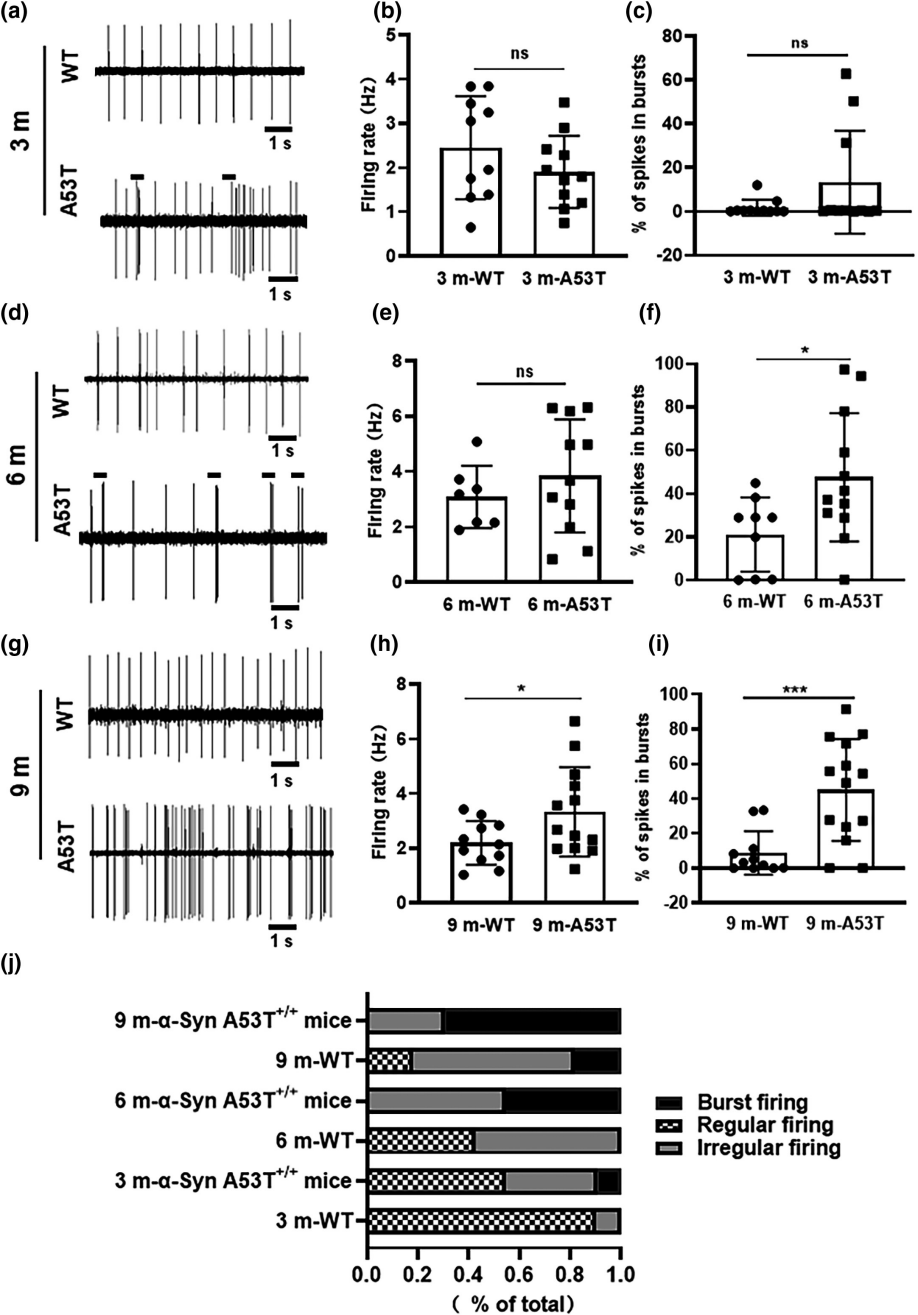
Spontaneous firing activities of dopaminergic neurons in the SN of α‐SynA53T^+/+^ mice at different ages. (a, d, g) The original spikes of both WT and α‐SynA53T^+/+^ mice in 3‐, 6‐, and 9‐month, respectively. (b, e, h) The summarized data showing the spontaneous firing rate of dopaminergic neurons in SN of both WT and α‐SynA53T^+/+^ mice in 3‐, 6‐, and 9‐month, respectively. (c, f, i) The summarized data showing the proportion of spikes in bursts of both WT and α‐SynA53T^+/+^ mice in 3‐, 6‐, and 9‐month, respectively. (j) The summarized data showing the percentage of the three firing patterns in WT and α‐SynA53T^+/+^ mice in 3‐, 6‐, and 9‐month (^*^ *p* < 0.05,^***^ *p* < 0.001)

One important function of dopaminergic neurons is their firing activity, which mediates the release of dopamine and controls motor behavior (Tsai et al., 2009). The spontaneous firing activity of nigral dopaminergic neurons exhibits specific features. Dopaminergic neurons have an obvious biphasic action potential with a long duration and a notch during the ascending phase, accompanied by a low frequency (<8 Hz) (Grace & Bunney, 1984b). GABAergic neurons in the SN have a higher firing rate (>10 Hz) than dopaminergic neurons and a shorter action potential duration without a notch (Wilson et al., 1977), as shown in Figure S1. However, the exact mechanism by which SUR1 participates in modulating the firing activity of dopaminergic neurons is unclear. To explore whether the firing activity changes with upregulation of SUR1, we observed the firing activity of dopaminergic neurons in the SN in α‐SynA53T^+/+^ mice at different ages. The spontaneous firing rate of dopaminergic neurons was not altered in 3‐month‐old α‐SynA53T^+/+^ mice (WT: 2.45 ± 1.16 Hz, A53T: 1.91 ± 0.81 Hz, *n* = 10,11, unpaired *t* test, *p *= 0.2243) and 6‐month‐old α‐SynA53T^+/+^ mice (WT: 3.08 ± 1.13 Hz, A53T: 3.84 ± 02.04 Hz, *n* = 7,11, unpaired *t* test, *p *= 0.3848) but was significantly increased by 51.75% in 9‐month‐old α‐SynA53T^+/+^ mice (WT: 2.53 ± 0.64 Hz, A53T: 3.74 ± 1.63 Hz, *n* = 8,10, unpaired *t* test, *p *= 0.0476) compared with that in the age‐matched WT mice (Figure 2a,b, d,e, and g,h). However, the proportion of spikes in bursts was significantly changed in 6‐month‐old α‐SynA53T^+/+^ mice (unpaired *t* test, *p *= 0.0281) and 9‐month‐old α‐SynA53T^+/+^ mice (unpaired *t* test, *p *= 0.0009) but not in 3‐month‐old α‐SynA53T^+/+^ mice (unpaired *t* test, *p *= 0.1196) (Figure 2c, f and i).

We refer to the criterion of dopaminergic neurons’ firing pattern defined by Grace and Bunney, which is explained in detail in our Methods section (Grace & Bunney, 1984a, 1984b). In our analysis of firing patterns (Figure 2j), in 3‐month‐old α‐SynA53T^+/+^ mice, 6 of the 11 (54.55%) dopaminergic neurons were identified as regular‐firing neurons, 4 of the 11 (36.36%) dopaminergic neurons were identified as irregularly discharging neurons, and 9% of the dopaminergic neurons were identified as cluster discharging neurons. In 6‐month‐old α‐SynA53T^+/+^ mice, 6 of the 11 (54.55%) dopaminergic neurons were identified as irregularly discharging neurons, and 5 of the 11 (45.45%) dopaminergic neurons were identified as cluster discharging neurons. In 9‐month‐old α‐SynA53T^+/+^ mice, 9 of the 13 (69.23%) dopaminergic neurons were identified as cluster discharging neurons. Consistent with the analysis of the proportion of spikes in bursts, the percentage of cluster discharging neurons was increased in 6‐month‐old α‐SynA53T^+/+^ mice (chi‐square, *p *= 0.013) and 9‐month‐old α‐SynA53T^+/+^ mice (chi‐square, *p *= 0.010) compared with the age‐matched WT mice. These results indicated that the firing rate and firing pattern of dopaminergic neurons changed with age, as especially evident in 9‐month‐old α‐SynA53T^+/+^ mice.

Moreover, in order to verify if the changes of the firing activity were affected by the SUR1, we also observed the spontaneous firing frequency and the proportion of spikes in bursts after α‐Syn knockdown in mice. As shown in Figure S2a–c, the spontaneous firing frequency of A53T‐α‐Syn‐KD mice was not changed (A53T: 3.84 ± 0.62 Hz, A53T‐α‐Syn‐KD: 3.88 ± 0.36 Hz, *n* = 11,12, unpaired *t* test, *p *= 0.948) but the proportion of spikes in bursts was decreased significantly (A53T: 47.55 ± 8.56%, A53T‐α‐Syn‐KD: 6.99 ± 1.39%; *n* = 11,12; unpaired *t* test, *p *< 0.0001). In summary, the firing activity of dopaminergic neurons was affected in the progression of PD with α‐Syn overexpression.

### 2.3 The K_ATP_ channel inhibitor glibenclamide increased the frequency of spontaneous discharge of nigral dopaminergic neurons in mice at different ages

**FIGURE 3:**
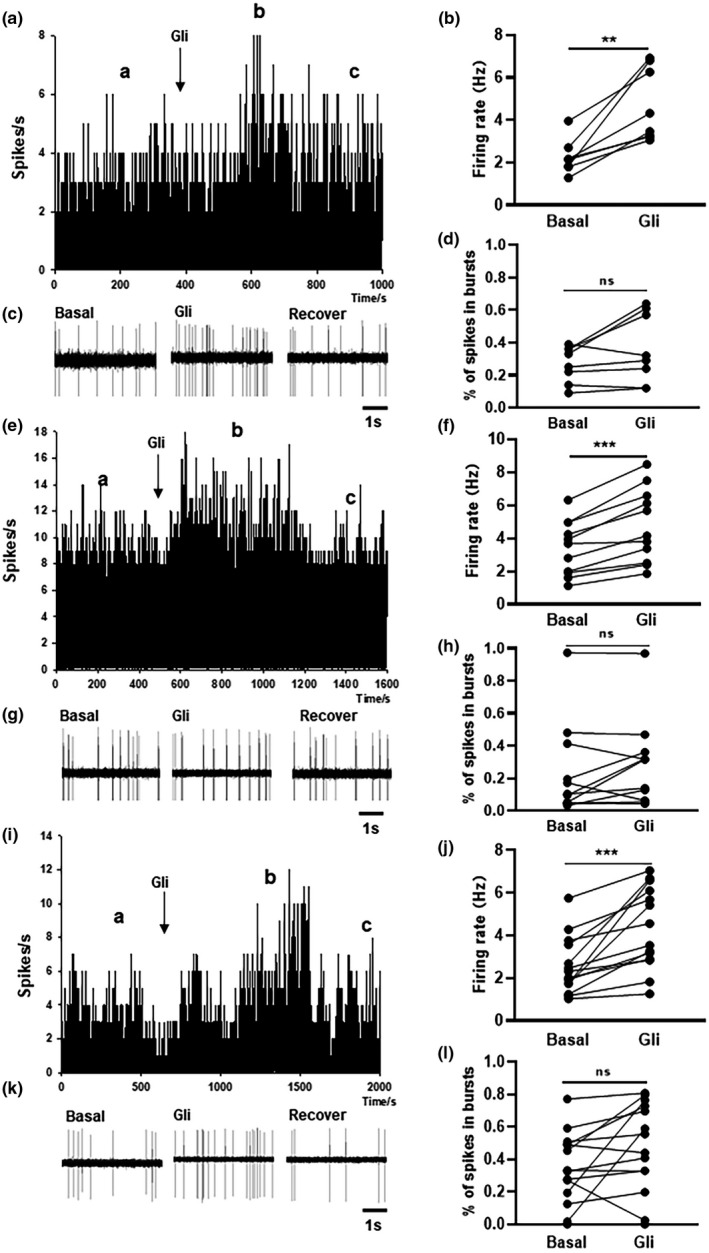
Effects of glibenclamide on spontaneous firing activities of dopaminergic neurons in the SN of α‐SynA53T^+/+^ mice at different ages. (a, e, and i) Typical frequency histogram showed that the firing rate of dopaminergic neurons was increased by glibenclamide in the SN in 3‐(a), 6‐ (e), and 9‐month α‐SynA53T^+/+^ mice (i). (c, g, and k) The original spikes at different stages in 3‐ (c), 6‐ (g), and 9‐month α‐SynA53T^+/+^ mice (k). (b, f, and j). The summarized data showing the effects of glibenclamide on the firing rate of dopaminergic neurons in the SN in 3‐, 6‐, or 9‐month α‐SynA53T^+/+^ mice. (d, h, and l). The summarized data showing the effects of glibenclamide on the proportion of spikes in bursts of dopaminergic neurons in the SN in 6‐ and 9‐month α‐SynA53T^+/+^ mice (^**^ *p* < 0.01, ^***^ *p* < 0.001)

To explore whether K_ATP_ channels intimately affect the activity of dopaminergic neurons in PD, we used glibenclamide as a K_ATP_ channel inhibitor in vivo. Firstly, in 3‐month‐old α‐SynA53T^+/+^ mice, the firing rate of nigral dopaminergic neurons was increased 106% after glibenclamide treatment (basal: 2.26 ± 0.80 Hz, glibenclamide: 4.66 ± 1.70 Hz; *n* = 8; paired *t* test, *p *= 0.0023), as shown in Figure 3a–c. The proportion of spikes in bursts did not change markedly after treatment with glibenclamide (basal: 26.75 ± 3.93%, glibenclamide: 36.38 ± 7.57%; *n* = 8; paired t test, *p *= 0.0908), as shown in Figure 3d. In 6‐month‐old α‐SynA53T^+/+^ mice, the spontaneous firing frequency in the 11 dopaminergic neurons was increased by 39.56% after glibenclamide treatment (basal: 3.41 ± 0.49 Hz, glibenclamide: 4.76 ± 0.67 Hz; paired *t* test, *p *= 0.0001), as shown in Figure 3e–g. However, glibenclamide treatment did not significantly change the proportion of spikes in bursts (basal: 23.69 ± 8.66%, glibenclamide: 28.87 ± 8.07%; paired *t* test, *p *= 0.1915), as shown in Figure 3h. In 9‐month‐old α‐SynA53T^+/+^ mice, the spontaneous firing frequency was increased from 2.56 ± 0.34 Hz to 4.31 ± 0.51 Hz, with an average increase of 70.65%, after treatment with glibenclamide (paired *t* test, *p *= 0.0004), as shown in Figure 3i–k. However, the change in the proportion of spikes in bursts was not statistically significant (basal: 34.71 ± 5.89%, glibenclamide: 47.68 ± 7.35%; *n* = 14; paired t test, *p* = 0.0543), as shown in Figure 3l. In general, these findings indicate that this K_ATP_ channel indeed participates in the firing activity of dopaminergic neurons in the progression of PD.

### 2.4 SUR1 knockdown antagonized the degeneration of nigral dopaminergic neurons in α‐SynA53T^+/+^ mice

**FIGURE 4:**
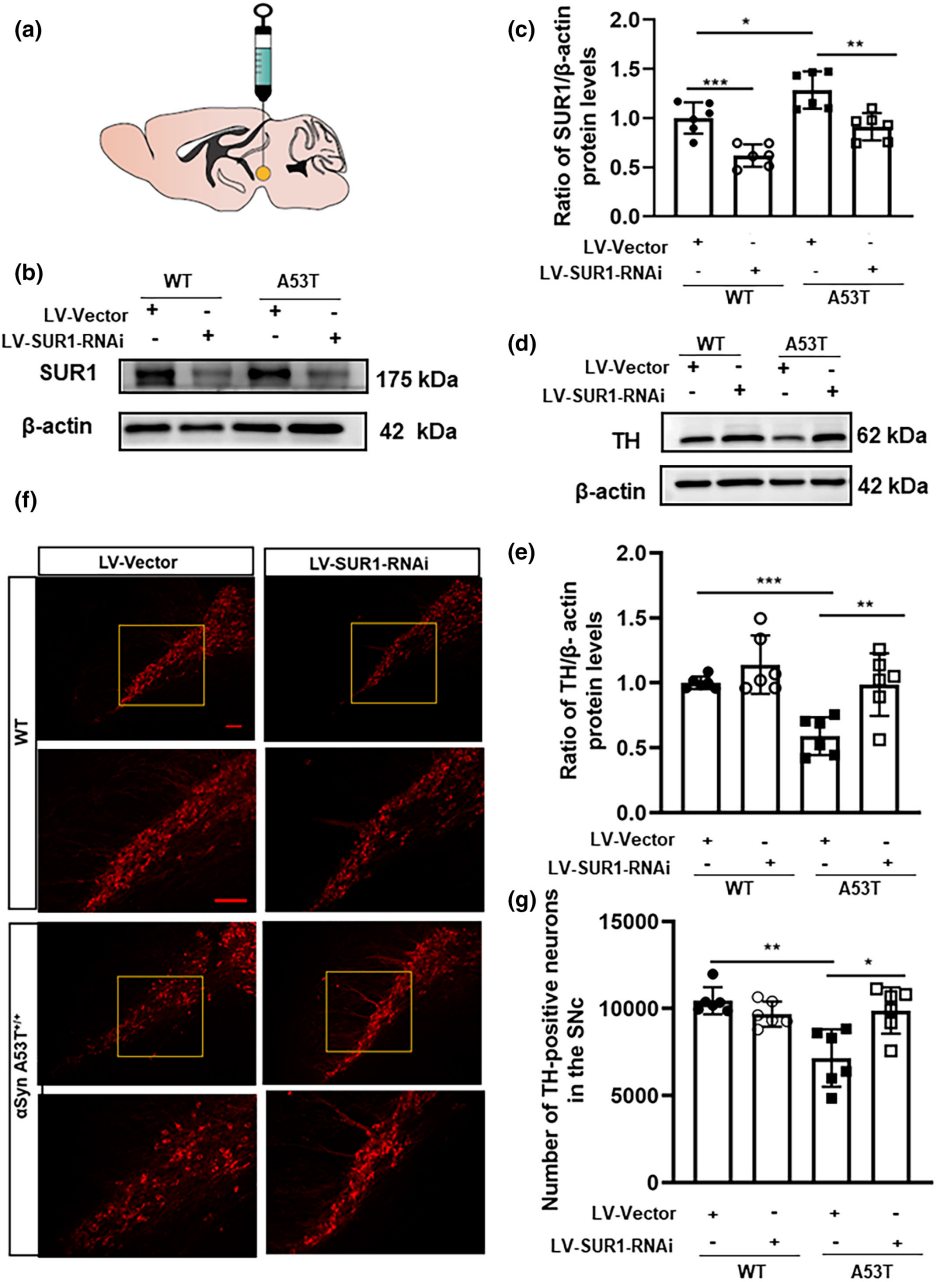
Effects of SUR1 knockdown on the progression of dopaminergic neurons degeneration in the SN of α‐SynA53T^+/+^ mice. (a). Schematic diagram of virus injection location. (b, c). The SUR1 protein levels of α‐SynA53T^+/+^ mice with LV‐SUR1‐RNAi in the SN were decreased, compared with the control group. (d, e). The change of TH protein levels after SUR1 knockdown in α‐SynA53T^+/+^ mice. (f, g). The change of the number of TH‐positive neurons after SUR1 knockdown in α‐SynA53T^+/+^ mice (^*^ *p *< 0.05, ^**^ *p *< 0.01). Scale bar = 100 μm

To further explore the role of the SUR1 subunit in the degeneration of nigral dopaminergic neurons, lentiviruses containing the SUR1 siRNA vector or the empty lentiviral vector were injected stereotaxically (Figure 4a) into the SN of 4‐month‐old WT mice and α‐SynA53T^+/+^ mice. In order to increase the efficiency of the infection, these mice were not used until 8 weeks after the virus injection in the SN. After injection of SUR1 knockdown lentivirus, the SUR1 protein levels were decreased by 38.25% (Tukey's multiple comparisons test, *p* = 0.0336) and 29.08% (Tukey's multiple comparisons test, *p* = 0.0310) in WT and α‐SynA53T^+/+^ mice, respectively (Figure 4b,c). In addition, the SUR1 protein levels in α‐SynA53T^+/+^ mice injected with empty lentivirus were increased by 28.47% compared with that in WT mice injected with empty lentivirus (Tukey's multiple comparisons test, *p *= 0.0464) (Figure 4b,c). As shown in Figure 4d–g, the protein level of tyrosine hydroxylase (TH) and the number of TH‐positive neurons in the SN in α‐SynA53T^+/+^ mice injected with empty lentivirus were decreased by 41.05% (Tukey's multiple comparisons test, *p *< 0.001) and 31.55% (Tukey's multiple comparisons test, *p *= 0.0013), respectively, compared with WT mice injected with empty lentivirus. The protein expression of TH and the number of TH‐positive neurons in the SN were increased by 67.41% (Tukey's multiple comparisons test, *p *= 0.0061) and 38.18% (Tukey's multiple comparisons test, *p *= 0.0102), respectively, in α‐SynA53T^+/+^ mice injected with SUR1 knockdown lentivirus compared with α‐SynA53T^+/+^ mice injected with empty lentivirus. These results demonstrated that the overexpression of SUR1 should be a risk factor in the progression of PD, and the degeneration of dopaminergic neurons would be delayed after SUR1 knockdown.

### 2.5 FOXA1 and FOXA2 could act as transcription factors to regulate the expression of the SUR1 subunit

**FIGURE 5:**
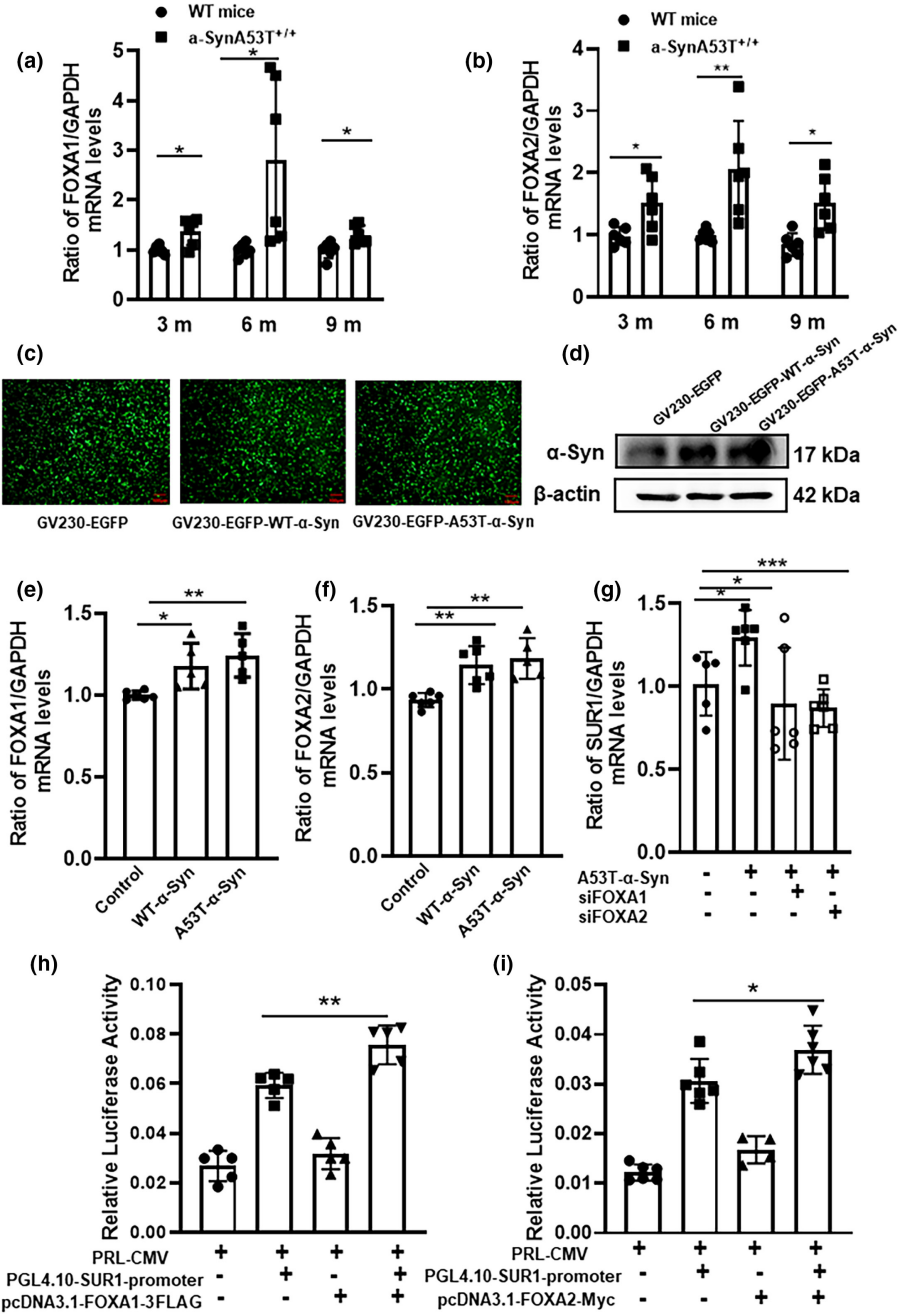
Changes of FOXA1 and FOXA2 expression levels in the SN of α‐SynA53T^+/+^ mice at different ages. (a, b) The mRNA levels of FOXA1 (a) and FOXA2 (b) of 3‐,6‐, and 9‐month α‐SynA53T^+/+^ mice in the SN. The house‐Keeping gene, GAPDH, was served as the standardized control. (c, d). Representational image of plasmid transfection and change of expression levels about α‐Syn after transfected successfully. (e, f). The mRNA expression of FOXA1 (e) and FOXA2 (F) after MES23.5 cells transfected with WT‐α‐Syn and A53T‐α‐Syn for 24 h. The house‐Keeping gene, GAPDH, was served as the standardized control. (g) Change of the mRNA levels of SUR1 in A53T α‐Syn group after the MES23.5 cell transfected with siFOXA1 + A53T‐α‐Syn or siFOXA2 + A53T‐α‐Syn for 24 h. (h, i) Dual luciferase results showed that the firefly/renilla luciferase activity in the SUR1‐FOXA1 group (h) and SUR1‐FOXA2 group (i) (^*^ *p* < 0.05, ^**^ *p* < 0.01, ^***^ *p* < 0.001)

To verify whether the upregulation of SUR1 expression was related to FOXA1 and FOXA2, we first examined the alterations in the mRNA levels of FOXA1 and FOXA2 in the SN in α‐SynA53T^+/+^ mice. As shown in Figure 5a,b, the mRNA levels of FOXA1 and FOXA2 in the SN in 3‐month‐old α‐SynA53T^+/+^ mice were increased by 37.27% (unpaired *t* test, *p *= 0.0101) and 153% (unpaired *t* test, *p *= 0.0206), respectively, compared with those in the age‐matched WT mice. In 6‐month‐old α‐SynA53T^+/+^ mice, the mRNA levels of FOXA1 and FOXA2 in the SN were increased by 180% (unpaired *t* test, *p *= 0.0233) and 204% (unpaired *t* test, *p *= 0.0083), respectively, compared with those in the age‐matched WT mice. In 9‐month‐old α‐SynA53T^+/+^ mice, the mRNA levels of FOXA1 and FOXA2 in the SN were increased by 130% (unpaired *t* test, *p *= 0.0130) and 179% (unpaired *t* test, *p *= 0.0060), respectively, compared with those in the age‐matched WT mice.

We also detected the expression of the transcription factors FOXA1 and FOXA2 by RT–PCR after overexpression of WT‐α‐Syn and A53T‐α‐Syn in MES23.5 cells (Figure 5c,d). As shown in Figure 5e,f, the mRNA levels of FOXA1 in the WT‐α‐Syn and A53T‐α‐Syn groups were significantly increased by 36% (Tukey's multiple comparisons test, *p *= 0.0422) and 49% (Tukey's multiple comparisons test, *p *= 0.0068), respectively. The mRNA levels of FOXA2 in the WT‐α‐Syn group and A53T‐α‐Syn group were increased by 35% (Tukey's multiple comparisons test, *p *= 0.0061) and 41% (Tukey's multiple comparisons test, *p *= 0.0023), respectively. After transfection of the siFOXA1 and A53T‐α‐Syn plasmids, as well as the siFOXA2 and A53T‐α‐Syn plasmids, into MES23.5 cells for 24 h, RT–PCR was performed. The mRNA levels of the SUR1 subunit in the A53T‐α‐Syn group were increased by 29% (Tukey's multiple comparisons test, *p *= 0.0300) compared with that in the control group (*p *< 0.05) (Figure 5g). The mRNA levels of the SUR1 subunit in cells transfected with siFOXA1 + A53T‐α‐Syn and siFOXA2 + A53T‐α‐Syn were decreased by 31% (Tukey's multiple comparisons test, *p *= 0.0273) and 33% (Tukey's multiple comparisons test, *p *= 0.0004), respectively, compared with that in MES23.5 cells transfected with only A53T‐α‐Syn (*p *< 0.05, *p *< 0.001).

Then, we performed a dual‐luciferase reporter assay to verify whether SUR1 was regulated by FOXA1 and FOXA2 (Figure 5h,i). When the pGL4.10‐SUR1 promoter plasmid and pcDNA3.1(+)‐FOXA1‐3FLAG plasmid were co‐transfected into HEK293 cells, the relative luciferase activity was increased by 27.5% (Tukey's multiple comparisons test, *p *= 0.0049) compared with that in cells co‐transfected with the pGL4.10‐SUR1 promoter and pcDNA3.1‐3FLAG plasmids. Moreover, when the pGL4.10‐SUR1 promoter plasmid and pcDNA3.1(+)‐FOXA2‐Myc plasmid were co‐transfected into HEK293 cells, the relative luciferase activity was increased by 20.5% (Tukey's multiple comparisons test, *p *= 0.0423), compared with that in cells co‐transfected with the pGL4.10‐SUR1 promoter and pcDNA3.1‐Myc plasmids. These results suggested that the transcription factors FOXA1 and FOXA2 could directly bind to the promoter of SUR1. However, FOXA1 and FOXA2 could not target the SUR2B or Kir6.2 promoters, as shown in S‐Figure 4a–d.

## 3 DISCUSSION

In the present study, we demonstrated that FOXA1 and FOXA2 were the causes of upregulation of SUR1 subunit in α‐Syn overexpressed models in vivo and in vitro. Moreover, the changes in electrical activities regulated by K_ATP_ channels play an important role in the progression of dopaminergic neurons degeneration in PD.

We previously demonstrated that the dopamine transporter (DAT) levels in α‐SynA53T^+/+^ mice were decreased at the ages of 6, 9, and 12 months by ^11^C‐2β‐carbomethoxy‐3β‐(4‐fluorophenyl) tropane positron emission tomography (^11^C‐CFT PET), indicating that the ability of DAT in dopaminergic neurons was reduced. However, the DAT levels were not significantly decreased in 3‐month‐old α‐SynA53T^+/+^ mice (Wang et al., 2018). The decreased DAT binding ability was associated with the progression of PD patients (Ikeda et al., 2019; Ishibashi et al., 2014). According to the previous studies, we examined the expressions of K_ATP_ channels subunits in α‐SynA53T^+/+^ mice at the ages of 3, 6, and 9 months. It is noteworthy that the expression of the SUR1 subunit has been increased in 3‐month‐old α‐SynA53T^+/+^ mice, even though the mice did not show significant loss of dopaminergic neurons at that age. A higher level of SUR1 expression has been reported to promote the K_ATP_ channels assembling and cell membrane trafficking, enhance the open abilities of the channels on cell surface (Yan et al., 2010).

General consensus is that K_ATP_ channels could control the neuronal excitability, energy metabolism, and neurotransmitter release (Mollajew et al., 2013; Nelson et al., 2015; Nguyen et al., 2019). But in different ages of α‐SynA53T^+/+^ mice, we only observed changes in the autonomous firing rate in 9‐month‐old α‐SynA53T^+/+^ mice, and we did not observe a significant difference in the autonomous firing rate in 3‐ or 6‐month‐old α‐ SynA53T^+/+^ mice. Moreover, the addition of glibenclamide could increase the firing rate in 3‐ and 6‐month‐old α‐SynA53T^+/+^ mice. Consistent with our study, Lasser‐Katz et al showed a twofold higher mean firing rate accompanied by an increase in abnormal aggregation of α‐Syn in the SN in middle‐aged (6–10 months) α‐SynA53T^+/+^ mice in vivo (Lasser‐Katz et al., 2017), and Subramaniam et al suggested that α‐Syn increases the firing frequency of nigral dopaminergic neurons via oxidative impairment of A‐type potassium channels (Subramaniam et al., 2014). But (Hill et al., 2021) demonstrated that α‐Syn aggregates increased whole‐cell conductance and decreased the firing rate in single dopaminergic neurons in the SN, and these effects were diminished by inhibition of K_ATP_ channels. This discrepancy might arise from the difference of the pathogenesis mechanism between α‐Syn oligomers treatment in vitro and mutant α‐Syn overexpression in α‐SynA53T^+/+^ mice; alternatively, the regulation of circuits in vivo could also be a cause. Therefore, we hypothesized that the different results in α‐SynA53T^+/+^ mice at different ages might be caused by the balance of mutant α‐Syn overexpression and the function of K_ATP_ channels. α‐Syn overexpression could induce the hyperactivity of nigral dopaminergic neurons through Ca^2+^ influx via Cav channels (Dryanovski et al., 2013; Lasser‐Katz et al., 2017), which induces local ADP accumulation, and then activates K_ATP_ channels (Knowlton et al., 2018). However, the opening of K_ATP_ channels could induce hyperpolarization of the cell membrane and decrease the autonomous firing rate by inhibiting Ca^2+^ flux. A similar phenomenon has been observed in hippocampal neurons, suggesting that K_ATP_ channels could dampen the enhanced neuronal excitability induced by the formation of nonselective cation channels by α‐Syn (Mironov, 2015). Therefore, in the early stage of PD, like in 3‐ and 6‐month‐old α‐SynA53T^+/+^ mice, we did not observe any changes in the spontaneous firing rate of dopaminergic neurons, the K_ATP_ channels attempted to rectify the hyperactivity of dopaminergic neurons induced by α‐Syn overexpression (Hill et al., 2021). However, in 9‐month‐old α‐SynA53T^+/+^ mice, the firing rate of the dopaminergic neurons began to increase with the gradual aggregation of α‐Syn.

But the effects of K_ATP_ channel activity might be a double‐edged sword in PD. Previous studies have indicated that the discharge pattern of remaining dopaminergic neurons in PD patients is changed from a tonic single peak discharge pattern to a cluster discharge pattern, and in which NMDA receptors and K_ATP_ channels are essential components (Dragicevic et al., 2015; Schiemann et al., 2012; Shen & Johnson, 2010; Shen et al., 2014). In our studies, we also verified that K_ATP_ channels participate in NMDA‐mediated burst firing in dopaminergic neurons in vivo in 3‐month‐old WT mice (as shown in Figure S3). α‐Syn could enhance the activation effect of Tau on non‐receptor tyrosine kinase Fyn by combining with Tau, inducing Fyn overexpression. Moreover, Fyn could phosphorylate the c‐terminal site Y1325 of NR2A receptor, which leads to the hyperactivation of NMDA receptors (Trudler et al., 2021). Therefore, the occurrence of burst firing should be partially blocked by the inhibition of K_ATP_ channels, but in α‐SynA53T^+/+^ mice, with α‐Syn overexpression induced high spontaneous firing rate, the occurrence of burst firing was not changed. However, α‐Syn knockdown may affect the expression or the function of K_ATP_ channels and NMDA receptors at the same time. Therefore, we observed the occurrence of burst firing was decreased significantly after α‐Syn knockdown in the present study (as shown in Figure S2a,b). The hyperexcitability and burst firing of neurons could promote DA release in the short term, but long‐term high‐frequency firing would lead to intracellular Ca^2+^ overload (Beatty et al., 2012; Shen & Johnson, 2010). Even worse, this vicious circle could eventually induce the degeneration of dopaminergic neurons. To further verify the role of K_ATP_ channels in the degeneration of dopaminergic neurons in PD, lentivirus‐mediated interference with SUR1 subunit expression in the SN was accomplished in 4‐month‐old α‐SynA53T^+/+^ mice. After 2 months, both the TH protein expression level and the number of TH‐positive neurons were significantly increased, indicating that interference with SUR1 expression at a very early stage of PD can partially antagonize dopaminergic neuron degeneration and delay the progression of PD.

Although some regulatory factors of SUR1 subunit have been demonstrated, the underlying regulatory mechanism of SUR1 subunit in nigral dopaminergic neurons is still largely unknown. FOXA1 and FOXA2 have been proven to play an essential role in the generation of dopaminergic neurons during early and late stages of development (Ang, 2009; Lin et al., 2009). Although the synthesis and release of DA in the SN was decreased in FOXA1/2^−/−^ mice at 12 weeks of age, the number of TH‐positive neurons in FOXA1/2^−/−^ mice at 24 weeks of age did not change (Domanskyi et al., 2014; Pristera et al., 2015; Stott et al., 2013). FOXA1 and FOXA2 can also regulate the expression of the SUR1 subunit as transcription factors in β cells (Cirillo et al., 2002; Jackson et al., 2010). In the present study, the levels of FOXA1 and FOXA2 were significantly increased in 3‐, 6‐, and 9‐month‐old α‐SynA53T^+/+^ mice and in α‐Syn‐overexpressing cells. Furthermore, we observed that FOXA1 and FOXA2 directly bound to the promoter region of SUR1. FOXA1 and FOXA2 might be mediators regulating the upregulation of SUR1 expression in α‐Syn overexpression models. However, the relationships between α‐Syn and FOXA1/FOXA2 need further clarification.

In conclusion, the SUR1 subunit of K_ATP_ channels was upregulated at the early stage of PD, an effect that might be related to the transcription factors FOXA1 and FOXA2. The enhanced activity of K_ATP_ channels antagonized the increase in the autonomous firing rate induced by α‐Syn overexpression at the early age, but the effect of K_ATP_ channel activity was weakened with further aggregation of α‐Syn. In addition, burst firing was increased with upregulation of the SUR1 subunit, which finally promoted dopaminergic neuron degeneration. The present study might provide evidence for the role of the SUR1 subunit of K_ATP_ channels in the neurodegenerative progression of PD.

## 4 MATERIALS AND METHODS

### 4.1 Animals

The transgenic mice expressing A53T human α‐syn (α‐SynA53T^+/+^ mice) were originally obtained from Jackson Laboratory (JAX004479, USA). All the mice were housed in their cages with a 12 h light/dark cycle with free access to food and water. Male homozygous (3‐, 6‐, and 9‐month) α‐SynA53T^+/+^ transgenic mice and their wild type littermates were used for the following experiments. Animal experiments were carried out according to the guidelines of National Institutes of Health Guidelines for the Care and Use of Laboratory Animals. All protocols were approved by the Animal Ethics Committee of Qingdao University.

#### 4.1.1 Total RNA extraction and reverse‐transcription (RT)‐PCR

Total RNA of the α‐SynA53T^+/+^ mice in the SN was harvested using Trizol Reagent (Invitrogen, USA) and reversely transcribed into cDNA using a First Strand cDNA Synthetic Kit (Thermo Fisher, USA). The mRNA levels of different subunits of K_ATP_ channels were detected by quantitative PCR with SYBR Green reagents (Qiagen, Germany). The primer sequences used are as follows: SUR1 F = 5′‐CCCTAGCTGTGGTGTGCTACTTCA‐3′, R = 5′‐GGGGCTGCGTTGTGTCATC‐3′; SUR2B F=5′‐TGGAGCTGACAGACACGAACAAC‐3′, R = 5′‐GAACAATGCACGCTCCCAGA‐3′; Kir6.2 F = 5′‐ATGGCCCTGACAGGCAAGAG‐3′, R = 5′‐CCAAGTTGGCCAGACAGACAGA‐3′; GAPDH F = 5′‐CAAATTCCATGGCACCGTCA‐3′, R = 5′ ATCGCCCCACTTGATTTTGG‐3′. The amplification was carried out according to the following steps: the mixture is preheated at 95°C for 30 s, followed by 5 s, 95°C and 34 s, 60°C for a total of 40 cycles. Relative mRNA levels were calculated using the 2^−ΔΔCt^ method.

#### 4.1.2 Western blots analysis

The SN of α‐SynA53T^+/+^ mice were homogenized by RIPA lysis buffer with 1% PMSF on ice for 30 min and then centrifugated at 15133 *g* for 20 min at 4°C. The supernatant was used for the following analysis. Protein concentration was determined with a BCA Assay Kit (Thermo Fisher, Germany). Lysates containing 60 μg of protein sample were separated on 8% (w/v) acrylamide gels and transferred to PVDF membranes with a diameter of 0.45 μm. After blocked with 10% non‐fat milk at room temperature for 1 h, the blots were incubated with anti‐SUR1 (1:1000, Abcam, UK), anti‐SUR2B (1:1000, Abcam, UK), TH (1:2000, Millipore, USA) and anti‐β‐actin (1:10,000, Santa Cruz, USA) overnight at 4°C. Membranes were incubated with HRP‐conjugated secondary antibodies (1:10,000, Santa Cruz, USA) for 1 h at room temperature. Finally, the antigen–antibody complexes were detected with enhanced chemiluminescence (Millipore, Billerica, MA, USA) and quantified with ImageJ Software.

#### 4.1.3 In vivo extracellular single unit electrophysiological recordings

The α‐SynA53T^+/+^ mice were anesthetized with urethane (1 g/kg, intraperitoneal injection) and positioned gently in the stereotaxic frame. A heating pad was used to maintain rectal temperature at 36–38°C. According to the stereotaxic atlas, a craniotomy was performed at coordinates of 3.00–3.30 mm posterior and 1.25–1.50 mm lateral from the bregma. Three‐barrel microelectrodes with a tip of 2–10 μm and a resistance of 10–20 MΩ were fastened by a pipette puller (Stoelting, Wood Dale, IL, USA). The electrodes were stereotaxically positioned into the SN. The recording barrel was filled with a commixture of .5 mol/L sodium acetate containing 2% pontamine sky blue. The other two micro‐pressure ejection barrels were connected to four‐channel pressure injector (PM2000B, Micro Data Instrument, Inc., USA), respectively, which contained (i) normal saline, (ii) glibenclamide (30 μmol/L) and vehicle (normal saline), (iii) NMDA (50 μmol/L) and vehicle (normal saline); (iv) glibenclamide and NMDA‐glibenclamide mixed solution (ratio 1:1). According to the location and electrophysiological features, the spontaneous firing neurons were identified as dopaminergic neurons. Drugs were ejected onto the surface of neurons with short pulse gas pressure (1500 ms, 5.0–15.0 psi). The recorded electrical signals were amplified by a microelectrode amplifier and displayed on a memory oscilloscope (VC‐11; Nihon Kohden, Japan). The amplified electrical signals were passed through low and high pass filters. Spike parameters were pre‐processed online and further analyzed offline using Spike 2 software (Cambridge Electronic Design Limited, Cambridge, UK) for spike data analysis. Drug infusion was performed only once for each recording and only one neuron was recorded in the same track. At least 5 min stable basal firing was collected from each neuron before drug ejection into dopaminergic neurons. The frequency of basal firing was determined by the average frequency of 120 s baseline data before drug administration. The maximal change of frequency within 50 s following drug application was considered as drug effects. A change in firing rate was considered to be significant if the change in firing rate exceeded two standard deviations. The firing patterns were identified based on the inter‐spike interval (ISI) histogram and auto‐correlogram analysis. The regular firing pattern was characterized by a normal distribution of ISI and at least three identifiable peaks in an auto‐correlogram. The irregular firing pattern showed a Poisson distribution of ISI and less than two peaks in an auto‐correlogram. The burst firing pattern displayed a positive skewed distribution of ISI and an auto‐correlogram with a single initial peak or without peak. The proportion of spikes in bursts was used to measure the burst firing. The onset of the burst was defined as the occurrence of two spikes with an ISI of <80 ms, and the termination of the burst defined as the‐occurrence of an ISI of 160 ms (Grace & Bunney, 1984b). After the electrophysiological study, the brains of mice were frozen and cut to examine the sites of recordings.

#### 4.1.4 Stereotactic injection

SUR1 interference lentivirus or α‐Syn interference was injected into the SN of α‐SynA53T^+/+^ mice with the following coordinates: anteroposterior (AP) = −3.2 mm, mediolateral (ML) = ±1.3 mm, and dorsoventral (DV) = −4.6 mm from bregma. SUR1 lentiviral vector and empty lentiviral vector were injected stereotaxically into the SN of 4‐month‐old WT mice and 4‐month‐old α‐SynA53T^+/+^ mice, as shown in Figure 4a. α‐SynA53T^+/+^ mice were randomly divided into four groups: WT + LV‐Vector group: WT mice were microinjected with LV‐Vector, α‐SynA53T^+/+^ + LV‐Vector group: α‐Syn A53T^+/+^mice were microinjected with LV‐Vector, WT + LV‐SUR1‐RNAi group: WT mice were microinjected with LV‐SUR1‐RNAi, α‐SynA53T^+/+^ + LV‐SUR1‐RNAi group: α‐SynA53T^+/+^ mice were microinjected with LV‐SUR1‐RNAi. The α‐Syn interference group was divided as same as the SUR1 interference group. Mice were deeply anesthetized with ether and decapitated. All mice were sacrificed for further investigation after 60 days. The brain was quickly removed and placed in ice‐cold artificial cerebrospinal fluid (ACSF) saturated with 95% O_2_ and 5% CO_2_ to maintain the pH = 7.4. The ACSF consisted of (in mM): 119 NaCl, 2.5 KCl, 11 glucose, 1.0 NaH_2_PO_4_, 26.2 NaHCO_3_, 2.5 CaCl_2_, and 1.3 MgCl_2_. Coronal slices (200 μm per each, 3 slices in total) containing the SN were cut with a microtome (Leica VT1200S). SN were microdissected in ice‐cold ACSF from the 200 μm tissue slices.

### 4.2 Immunofluorescent staining

Six‐month‐old mice with 50 mg/kg sodium pentobarbital anesthetized were perfused intracardially with phosphate buffer saline (PBS, 0.1 M, pH 7.2) followed by 4% (w/v) paraformaldehyde solution (PFA). Brains were removed and post‐fixed in PFA overnight at 4°C, then gradually transferred to 15% (w/v), 20% (w/v), and 30% (w/v) sucrose until sectioning. Sections (20 µm) were cut on a freezing microtome (CM1905, Leica, Germany). After being washed 3 times in PBST (0.3% Triton X‐100 in 0.01 M PBS), sections were blocked by 10% goat serum and then incubated overnight with primary antibody of TH (1:2000, Millipore, USA) at 4°C overnight. Then, the sections were rinsed three times with PBST and incubated in the second antibody of Alexa Fluor 488 donkey anti‐rabbit IgG at room temperature. Next, sections were mounted with 70% glycerin after rinsed with PBS for three times. The number of TH‐ immunoreactive (ir) neurons in the SN was estimated using MBF Stereo Investigator software. Experimenters were blinded to the specific information of each group.

#### 4.2.1 Cell culture and treatment

The rodent MES23.5 cell line was obtained from Dr. Wei‐dong Le at Dalian Medical University (Dalian, China). MES23.5 cells were cultured in Dulbecco's modified Eagle's medium (DMEM)‐ F12 (Gibco, USA) containing Sato's components growth medium supplemented with 5% fetal bovine serum, 100 units/mL of penicillin, and 100 units/mL of streptomycin in a humidified atmosphere containing 5% CO_2_ at 37°C. For experiments, cells were seeded at a density of 1 × 10^5^/cm^2^ in plates and grown to 70%–80% confluency. MES23.5 cells with A53T mutant α‐syn over expressing were cultured in six‐well plates at a density of 1 × 10^5^/cm^2^ in plates and grown to 70%–80% confluency for 24 h. The cells were separated to three groups: GV230 vector group, WT α‐Syn group, and A53T α‐syn group. In the control group, GV230 vectors were transfected into MES23.5 cells alone. In the over‐expression group, cells were transfected with the WT‐α‐syn or A53T‐α‐syn in a serum‐free medium. This relatively high efficiency of infection of MES23.5 cells was harvested for the further studies. All vectors were transfected using Lipofectamine 2000. For optimal transfection efficiency, five volumes of DNA relative to lipofectamine 2000 were used.

HEK293 cells’ culture method is similar to MES23.5 cells. HEK293 cells were transfection with Lipofectamine^™^ 2000, and the double luciferase reporter gene assay (Promega, E1910) was performed 24 h later. The relative fluorescence intensity was calculated using firefly fluorescence value/renin fluorescence values.

## CONFLICT OF INTEREST

The authors declare that the research was conducted in the absence of any commercial or financial relationships that could be construed as a potential conflict of interest.

## AUTHOR CONTRIBUTIONS

Hong Jiang and Xi‐xun Du conceived and designed research. Min Liu, Cui Liu, Xue Xiao, and Shuai‐shuai Han performed experiments and analyzed data. Liu Min wrote the manuscript. Hong Jiang, Xi‐xun Du, Qian Jiao, Mingxia Bi, Xi Chen, and Chun‐ling Yan revised the manuscript, and we thank Hong‐lin Zhu for assistance.

## Supporting information

Figure S1

Figure S2

Figure S3

Figure S4

## Data Availability

The data that support the findings of this study are available upon request.
