# Supplementary figures and images for: Role of upregulation of the KATP channel subunit SUR1 in dopaminergic neuron degeneration in Parkinson’s disease
Source: Aging Cell. 2022 Apr 20;21(5):e13618. doi: 10.1111/acel.13618 (PMC9124303; doi:10.1111/acel.13618)

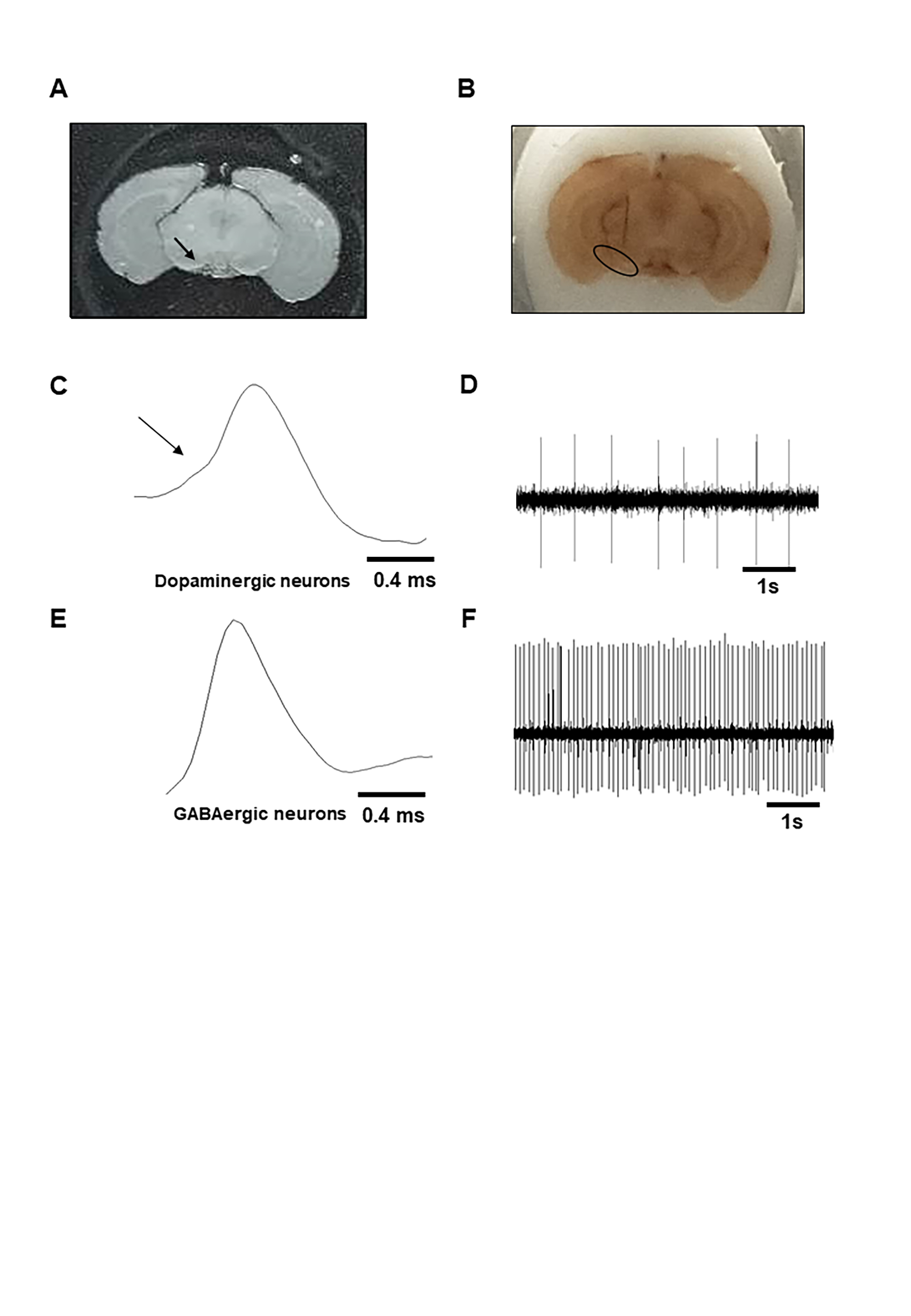

Supplement: Supplementary file 1 — Figure S1 [file ACEL-21-e13618-s004.jpg]

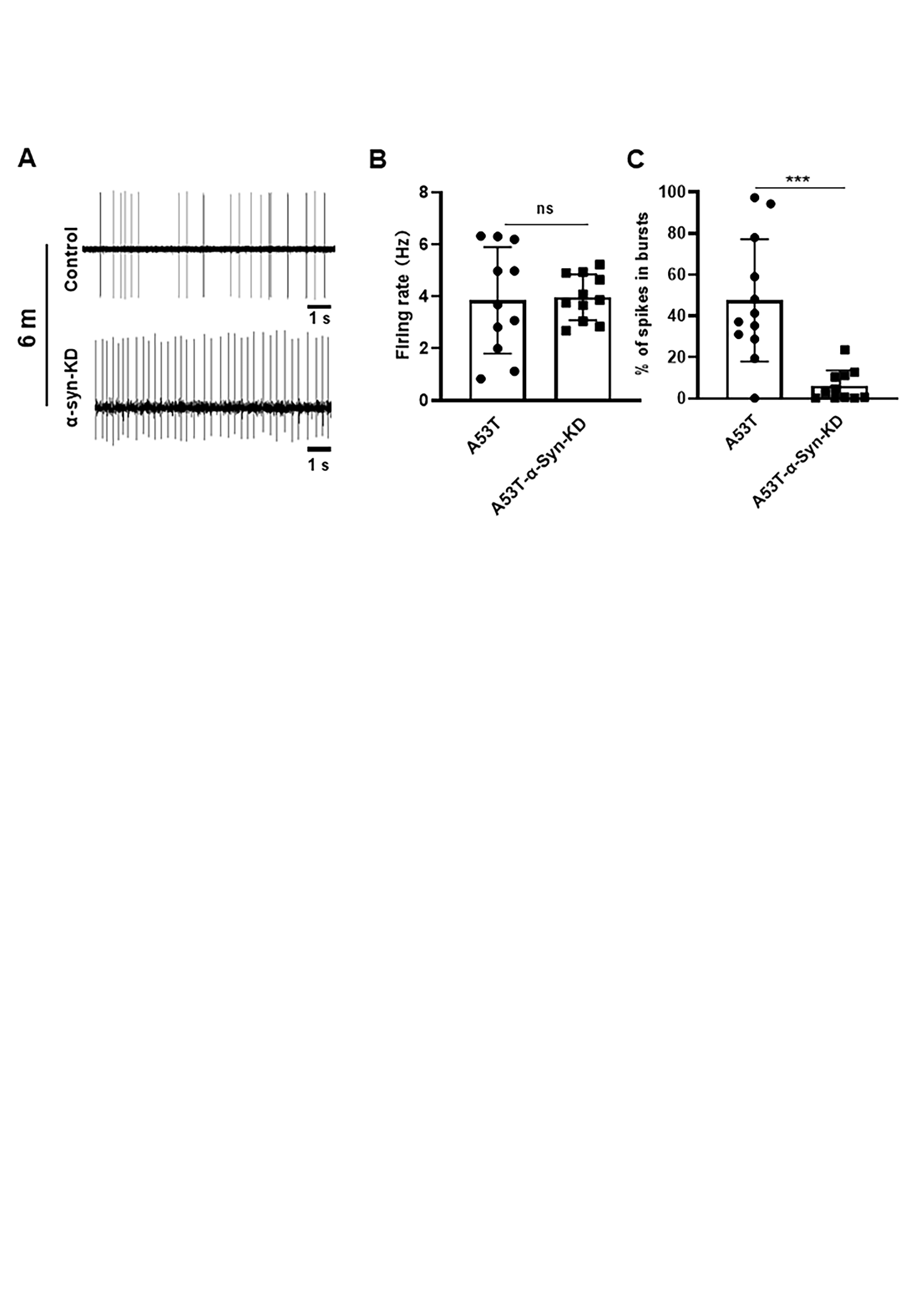

Supplement: Supplementary file 2 — Figure S2 [file ACEL-21-e13618-s002.jpg]

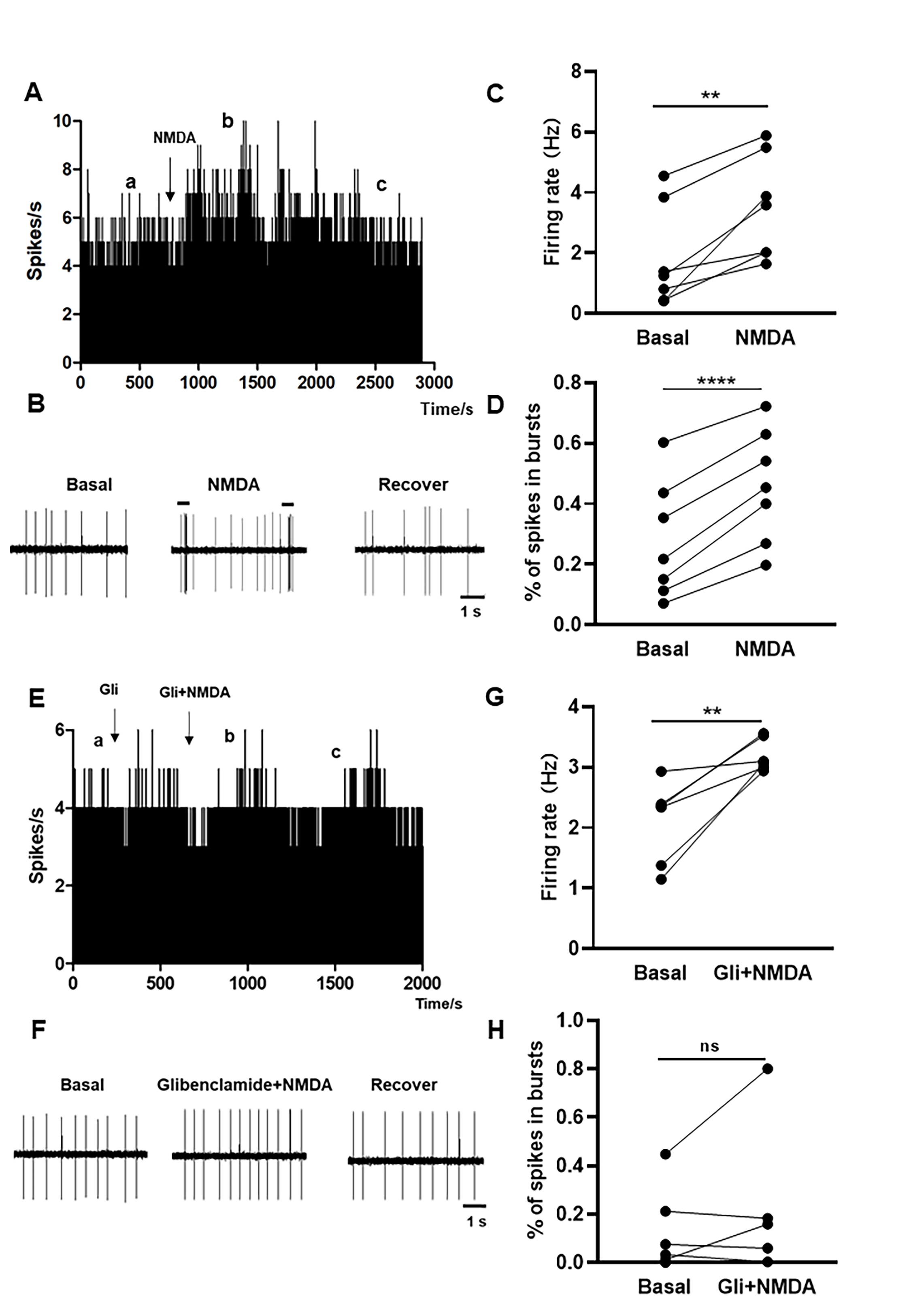

Supplement: Supplementary file 3 — Figure S3 [file ACEL-21-e13618-s003.jpg]

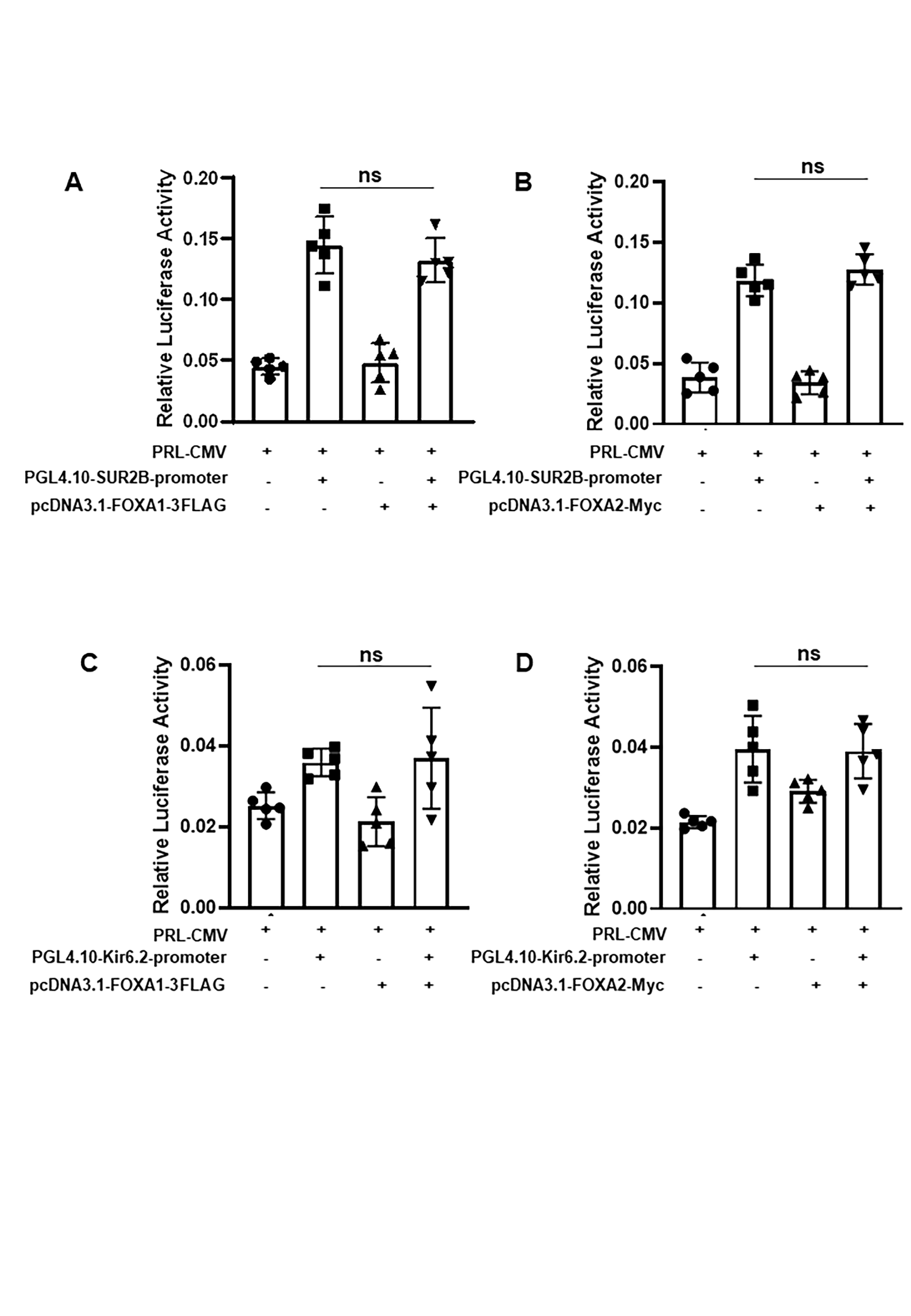

Supplement: Supplementary file 4 — Figure S4 [file ACEL-21-e13618-s001.jpg]
